# The association of central and extremity circumference with all-cause mortality and cardiovascular mortality: a cohort study

**DOI:** 10.3389/fcvm.2023.1251619

**Published:** 2023-08-31

**Authors:** Jiajun Liu, Xueshan Jin, Ziyi Feng, Jieming Huang

**Affiliations:** ^1^ Department of Gynecology, The First Affiliated Hospital of Guangzhou University of Chinese Medicine, Guangzhou, China; ^2^ First Clinical Medical College, Guangzhou University of Chinese Medicine, Guangzhou, China; ^3^ Nephropathy Center, The Affiliated Jiangmen TCM Hospital, Jinan University, Jiangmen, China

**Keywords:** central obesity, extremity circumference, waist circumference, arm circumference, calf circumference, thigh circumference, all-cause mortality, cardiovascular mortality

## Abstract

**Background:**

Central obesity increases the risk of several diseases, including diabetes, cardiovascular disease (CVD), and cancer. However, the association between extremity obesity and mortality has not been extensively evaluated. The objective of this study was to investigate the quantitative effects of waist circumference (WC), arm circumference (AC), calf circumference (CC), and thigh circumference (TC) on all-cause mortality and CVD mortality.

**Methods:**

The study used data from the National Health and Nutrition Examination Survey (NHANES) sample survey from 1999 to 2006. A total of 19,735 participants were included in the study. We divided the participants into four groups (Q1–Q4) and used Q1 as a reference to compare the risk of all-cause mortality and CVD mortality in Q2–Q4. COX proportional hazard regression model was used to analyze the relationship between WC, AC, CC and TC on all-cause and CVD mortality. In addition, we conducted a stratified analysis of gender.

**Results:**

After a mean follow-up of 11.8 years, we observed a total of 3,446 deaths, of which 591 were due to cardiovascular disease. The results showed that for both men and women, compared to the first group, the risk of all-cause mortality was significantly higher in the other three groups of WC and significantly lower in the other three groups of AC, CC, and TC. Similar results were observed after adjusting for confounding factors such as demographics.

**Conclusions:**

Our results show that all-cause and CVD mortality are positively associated with measures of central obesity and negatively associated with measures of extremity obesity, and that AC, CC, and TC can be used as potential tools to measure prognosis in the general population.

## 1. Introduction

Obesity is one of the major risk factors for noncommunicable diseases and is often associated with multiple comorbidities, including cardiovascular disease (CVD), diabetes, and some cancers (1, 2). Depending on the severity of comorbidities, obesity is estimated to reduce life expectancy by 5–20 years (3). Over the past half century, the prevalence of obesity has reached epidemic proportions (1, 4). Globally, the prevalence of obesity (BMI ≥30 kg/m^2^) increased from 3.2% to 10.8% in males and from 6.4% to 14.9% in females (5). In 2014, the prevalence of morbid obesity (BMI ≥40 kg/m^2^) was 0.64% in men and 1.6% in women, respectively. Therefore, it is important to develop clinical and public health strategies to prevent this chronic disease. Many indicators have been developed to predict the risk of obesity-related diseases. Anthropometric parameters are often used in clinical and epidemiological assessments of obesity and health status because they are accessible, inexpensive, and facilitate long-term monitoring (6–8). These parameters mainly include BMI, waist circumference (WC), and waist-to-height ratio (WHtR), all of which can be used to assess the risk associated with overall obesity or abdominal obesity (9, 10). A recent meta-analysis reported that obesity as defined by BMI was associated with lower all-cause mortality (11). Results from large observational studies also show that obesity as defined by WC is associated with total mortality, while obesity as defined by BMI is not, potentially challenging the paradigm that BMI is associated with increased mortality (12, 13).

Recent published studies have reported that an increase in WC may be associated with higher CVD mortality in older adults regardless of baseline WC level (14). A meta-analysis of 72 prospective cohort studies showed that most parameters of central obesity, including WC, WHtR, and waist-to-hip ratio (WHR), were significantly positively associated with a higher risk of all-cause mortality (15). This association remained significant after adjusting for BMI. After excluding measures of central obesity, several measures of extremity circumference were used to assess the risk of death associated with obesity. A prospective cohort study of 375 patients from 7 hospitals reported that higher mid-arm circumference was associated with lower mortality in hemodialysis patients (HR: 0.90, 95% CI: 0.82–0.99) (16). Similar results were reported in another study, which found that in people with diabetes, the risk of all-cause mortality and CVD mortality was significantly negatively associated with either the AC or arm-to-waist ratio (AC/WC) (14). The larger the AC or AC/WC, the stronger its protective effect on the body. Compared with AC, AC/WC is a better predictor of mortality risk in diabetes patients, because the increase of AC may be the result of obesity, and AC/WC may represent the standard body shape to some extent. However, few studies have investigated the association between thigh circumference (TC) or calf circumference (CC) and the risk of CVD mortality or all-cause mortality.

The aim of this study was to assess the association between central circumference, including WC, and extremity circumference, including AC, CC, TC, and all-cause or CVD mortality in the normal population, and to obtain quantitative estimates by regression analysis. We hypothesized that larger central circumference would increase all-cause mortality and CVD mortality, whereas larger extremity circumference would decrease both.

## 2. Methods

### 2.1. Data source

National Health and Nutrition Examination Survey (NHANES) is a large cross-sectional study conducted by the Centers for Disease Control and Prevention to assess the nutritional health status and morbidity of the United States population. The study conducts the survey every 2 years, using a complex multi-stage sampling to ensure that the survey population is representative. The survey mainly includes five parts: population data, diet data, examination data, laboratory data, and questionnaire data.

### 2.2. Study population

The study used data from the NHANES sample survey from 1999 to 2006. Participants were considered for inclusion if they met the following criteria: (1) Age of enrollment was ≥18 years old. (2) With available data about all-cause mortality and CVD mortality. (3) With available data about anthropometric parameters. Participants who had CVD at the time of enrollment were excluded. In addition, we also excluded deaths that occurred within 6 months after enrollment due to certain diseases, as these diseases are usually unrelated to obesity status. Individuals lacking key covariates, such as age and hypertension status, were excluded. Finally, a total of 19,735 participants were included in the study.

### 2.3. Measurement of anthropometric parameters

WC was measured in a standing position with the abdomen relaxed. When the participant is in the stage of minimal breathing, the operator wraps a tape measure around the iliac spine. During AC measurement, participants took a standing position with the right arm hanging freely and the elbow relaxed naturally. The operator places the tape measure at the midpoint between the acromial process and the olecranon process behind the upper arm, gently presses it to the skin surface. The length of the tape around the upper arm is AC. When calf circumference (CC) was measured, participants sat with their right calf relaxed. Trained NHANES personnel place the tape measure perpendicular to the long axis of the calf, finding the location of the maximum perimeter, which is CC. TC was measured in a standing position with all weight on the left leg. The operator places the tape measure perpendicular to the long axis of the thigh, and the data recorded around the middle area of the thigh is TC. WC, AC, CC, and TC were all obtained by trained professionals, and the value was measured to the nearest 0.1 cm.

### 2.4. Outcome ascertainment

The primary outcome measures in this study were defined as all-cause mortality and CVD mortality. The participants’ current living conditions and specific date of death were collected through interviews with close family members. Cardiovascular death was determined according to the International Classification of Diseases Version 10 (ICD-10) code I00-I78.

### 2.5. Measurement of covariates

Covariates include age, sex, race/ethnicity, education, physical activity, smoking status, drinking status, hypertension status, and hyperlipidemia status. Race/ethnicity is divided into the following five categories: Mexican American, other Hispanic, Non-Hispanic White, Non-Hispanic Black, and other races. Education is divided into three categories: under high school, high school or some college, college graduate or above. Levels of physical activity were classified as active, insufficiently active and inactive. Smoking status was classified as current smoker, former smoker, and never smoker. Drinking status was classified as heavy, moderate, mild or never. Hypertension, hyperlipidemia, and diabetes mellitus were classified as present or absent based on self-report at baseline.

### 2.6. Statistical analysis

NHANES is a stratified, multi-stage, and complex sample design survey, so all statistical analyses in our study used appropriate sample weights associated with the NHANES data. Baseline characteristics of participants were presented as number and percentage (%) for categorical variables and as mean and standard error (SE) for continuous variables. All anthropometric parameters were divided into quartiles (Q1–Q4), and Q1 was used as a reference. COX proportional hazard models were used to assess the relationship between anthropometric parameters and CVD and all-cause mortality. The results are expressed by the hazard ratio (HR) and the corresponding 95% confidence interval (CI). Model 1 is a coarse model without adjusting for confounders. Model 2 was adjusted for potential confounders, including gender, race/ethnicity, education, physical activity, alcohol consumption, and smoking status. Although we considered metabolic cardiovascular risk factors related to obesity when constructing survival models, we did not adjust for these factors in the final estimation. Published evidence suggests that factors that modulate the causal pathway between obesity and death, such as high blood pressure, diabetes, may be inappropriate (12). All statistical analyses were performed using Stata (version 15.1) software.

## 3. Results

### 3.1. Study characteristics

A total of 19,735 participants were included in the final analysis, including 7,472 men (47.99%) and 10,263 (52.01%) women. Table 1 shows the demographic characteristics and clinical disease status of participants grouped by sex. Most of the participants in the study were non-Hispanic white, and most had more than a high school education. Smoking and drinking are more common in men. Male participants had similar rates of hypertension, diabetes, and hyperlipidemia compared to female participants. According to the WHO's criteria for defining central obesity, 3,602 male (48.21%) and 6,451 female participants (62.86%) had central obesity (17). The characteristics of anthropometric parameters as four categorical variables can be obtained in Table 2.

**Table 1:** Baseline characteristics of study participants.

Characteristic	Male	Female
Participants, *n* (%)	9,472 (47.99)	10,263 (52.01)
Age (Mean ± SE), year	46.20 ± 0.21	44.69 ± 0.20
BMI (Mean ± SE), kg/m^2^	27.61 ± 0.06	28.38 ± 0.07
WC (Mean ± SE), cm	98.19 ± 0.16	94.17 ± 0.15
AC (Mean ± SE), cm	33.36 ± 0.04	31.70 ± 0.05
CC (Mean ± SE), cm	38.59 ± 0.04	37.84 ± 0.05
TC (Mean ± SE), cm	52.99 ± 0.07	52.95 ± 0.08
Race/ethnicity, *n* (%)		
Mexican American	2,206 (23.29)	2,422 (23.60)
Other Hispanic	355 (3.75)	458 (4.46)
Non-Hispanic white	4,568 (48.23)	4,820 (46.96)
Non-Hispanic black	2,002 (21.14)	2,149 (20.94)
Other race	341 (3.59)	414 (4.03)
Education, *n* (%)		
Under high school	1,940 (20.48)	1,840 (17.93)
High school or some college	4,780 (50.46)	5,446 (53.06)
College graduate or above	2,752 (29.05)	2,977 (29.02)
Smoking, *n* (%)		
Current	2,823 (29.80)	2,222 (21.65)
Former	674 (7.12)	465 (4.53)
Never	5,975 (63.08)	7,576 (73.82)
Drinking, *n* (%)		
Heavy	4,709 (49.71)	2,957 (28.81)
Moderate	2,252 (23.78)	2,713 (26.43)
Mild or never	2,511 (26.51)	4,593 (44.76)
Physical activity level, *n* (%)		
Active	3,726 (39.34)	3,206 (31.24)
Insufficiently active	2,694 (28.44)	3,547 (34.56)
Inactive	3,052 (32.22)	3,510 (34.20)
Diabetes mellitus		
Yes	812 (8.57)	627 (6.11)
No	8,660 (91.43)	9,636 (93.89)
Hypertension, *n* (%)		
Yes	2,969 (31.34)	3,219 (31.36)
No	6,503 (68.66)	7,044 (68.64)
Hyperlipidaemia, *n* (%)		
Yes	3,469 (36.62)	3,502 (34.12)
No	6,003 (63.38)	6,761 (65.88)

Continuous variables are expressed by means and standard errors (SE), categorical variables are expressed in numbers (percentages).

BMI, body mass index; WC, waist circumference; AC, arm circumference; CC, calf circumference; TC, thigh circumference.

**Table 2:** The characteristic of anthropometric parameters as four categorical variables.

	Q1		Q2		Q3		Q4	
	Mean	Range	Mean	Range	Mean	Range	Mean	Range
WC	77.34	56.0–84.9	90.33	85.0–95.4	100.39	95.5–105.9	116.51	106.0–175.0
AC	26.66	17.0–29.0	30.64	29.1–32.1	33.71	32.2–35.4	38.95	35.5–61.1
CC	33.15	20.7–35.2	36.53	35.3–37.7	39.13	37.8–40.6	43.92	40.7–75.6
TC	44.78	25.1–48.0	50.12	48.1–52.1	54.35	52.2–56.8	62.67	56.9–100.0

BMI, body mass index; WC, waist circumference; AC, arm circumference; CC, calf circumference; TC, thigh circumference.

### 3.2. The association between anthropometric parameters and mortality

After a mean follow-up of 11.8 years, we observed a total of 3,446 deaths, of which 591 were due to CVD. Tables 3, 4 show quantitative estimates of WC, AC, CC, and TC and all-cause mortality and CVD mortality by COX regression analysis. The results showed that for both men and women, compared to the first group, the risk of all-cause mortality was significantly higher in the other three groups of WC and significantly lower in the other three groups of AC, CC, and TC. Similar results were observed after adjusting for confounding factors such as demographics.

**Table 3:** The association between WC, AC, CC and TC and all-cause mortality risks.

	Male		Female	
	Model 1	Model 2	Model 1	Model 2
WC				
Q1	1	1	1	1
Q2	1.45 (1.23–1.71)	1.16 (0.98–1.37)	1.47 (1.27–1.70)	1.24 (1.07–1.44)
Q3	2.07 (1.78–2.41)	1.58 (1.35–1.85)	1.74 (1.50–2.00)	1.37 (1.18–1.58)
Q4	2.31 (1.98–2.68)	1.70 (1.45–1.98)	1.48 (1.27–1.72)	1.15 (0.99–1.34)
AC				
Q1	1	1	1	1
Q2	0.63 (0.56–0.71)	0.58 (0.51–0.65)	0.96 (0.85–1.10)	0.83 (0.72–0.94)
Q3	0.44 (0.39–0.50)	0.39 (0.35–0.45)	0.92 (0.80–1.06)	0.75 (0.65–0.87)
Q4	0.35 (0.30–0.40)	0.31 (0.27–0.35)	0.77 (0.67–0.89)	0.65 (0.56–0.75)
CC				
Q1	1	1	1	1
Q2	0.60 (0.53–0.67)	0.58 (0.52–0.65)	0.61 (0.54–0.70)	0.61 (0.53–0.69)
Q3	0.43 (0.38–0.48)	0.42 (0.37–0.47)	0.49 (0.43–0.57)	0.46 (0.39–0.53)
Q4	0.33 (0.29–0.37)	0.31 (0.27–0.36)	0.37 (0.32–0.43)	0.34 (0.29–0.40)
TC				
Q1	1	1	1	1
Q2	0.45 (0.40–0.50)	0.46 (0.41–0.51)	0.52 (0.45–0.59)	0.53 (0.47–0.61)
Q3	0.29 (0.25–0.32)	0.30 (0.26–0.34)	0.45 (0.39–0.52)	0.44 (0.38–0.50)
Q4	0.22 (0.19–0.26)	0.24 (0.21–0.28)	0.33 (0.29–0.39)	0.31 (0.27–0.37)

The data is expressed in hazard ratio (HR) and the corresponding 95% confidence interval (CI).

WC, waist circumference; AC, arm circumference; CC, calf circumference; TC, thigh circumference.

Model 1: no covariates were adjusted. Model 2 adjusted: age, sex, race/ethnicity, education.

**Table 4:** The association between WC, AC, CC and TC and cardiovascular disease mortality risks.

	Male		Female	
	Model 1	Model 2	Model 1	Model 2
WC				
Q1	1	1	1	1
Q2	2.38 (1.54–3.69)	1.75 (1.13–2.72)	1.69 (1.14–2.51)	1.40 (0.95–2.08)
Q3	3.58 (2.37–5.42)	2.49 (1.64–3.78)	2.07 (1.41–3.05)	1.59 (1.08–2.35)
Q4	3.79 (2.51–5.73)	2.54 (1.67–3.86)	1.77 (1.18–2.64)	1.32 (0.88–1.98)
AC				
Q1	1	1	1	1
Q2	0.54 (0.41–0.72)	0.48 (0.37–0.64)	0.93 (0.67–1.30)	0.78 (0.56–1.10)
Q3	0.38 (0.29–0.51)	0.33 (0.24–0.44)	0.75 (0.51–1.11)	0.60 (0.41–0.89)
Q4	0.40 (0.30–0.53)	0.34 (0.25–0.46)	0.71 (0.49–1.03)	0.56 (0.38–0.82)
CC				
Q1	1	1	1	1
Q2	0.58 (0.45–0.76)	0.57 (0.44–0.74)	0.40 (0.27–0.58)	0.39 (0.27–0.57)
Q3	0.39 (0.30–0.52)	0.38 (0.28–0.51)	0.50 (0.35–0.72)	0.46 (0.32–0.66)
Q4	0.37 (0.27–0.49)	0.35 (0.26–0.47)	0.42 (0.29–0.62)	0.38 (0.26–0.56)
TC				
Q1	1	1	1	1
Q2	0.38 (0.30–0.50)	0.39 (0.30–0.50)	0.36 (0.24–0.53)	0.37 (0.25–0.54)
Q3	0.24 (0.18–0.33)	0.25 (0.19–0.34)	0.40 (0.28–0.59)	0.38 (0.26–0.56)
Q4	0.26 (0.19–0.35)	0.28 (0.20–0.38)	0.39 (0.27–0.55)	0.35 (0.24–0.50)

The data is expressed in hazard ratio (HR) and the corresponding 95% confidence interval (CI).

WC, waist circumference; AC, arm circumference; CC, calf circumference; TC, thigh circumference.

Model 1: no covariates were adjusted. Model 2 adjusted: age, sex, race/ethnicity, education.

## 4. Discussion

The cohort study included 19,735 participants and compared the association between one measure of central obesity (WC) and three measures of extremity obesity (AC, CC, and TC) with all-cause mortality and CVD mortality. The most prominent finding of this study was that increases in WC were associated with increased all-cause mortality and CVD mortality, while increases in AC, CC, and TC were associated with reduced all-cause mortality and CVD mortality. Our results confirm previous reports that central obesity is associated with an increased risk of premature death, and suggest that extremity obesity has a protective effect on the body.

Previous studies have shown that anthropometric parameters can be used to predict mortality risk. AC was as good at predicting a population's risk of death as other measures, including body mass index, age-specific body mass index, and weight Z score (18–20). A cohort study of 160 older adults showed that AC was stronger than BMI in predicting the risk of death within 12 months (21). Another nationally representative study from the Netherlands showed that among older residents of the community, low AC in both sexes was associated with a significantly increased risk of death within 15 years (22). This association remained after excluding deaths within 3 years of enrollment, or patients with cancer or chronic obstructive pulmonary disease. The authors further investigated and found that low AC was more strongly associated with 15-year mortality than low BMI. In older adults, BMI may be difficult to obtain, and simple anthropometry such as AC has a stronger association with mortality, so AC may be a potential indicator for predicting mortality. However, some cross-sectional studies have reported the opposite conclusion. Results from a cross-sectional study of preschool children in Africa suggest that a higher AC may be associated with an increased risk of cardiovascular risk factors, which is consistent with another study in China (23, 24). Recently published evidence suggests that increased CC may be associated with a better prognosis. A cohort study from an Asian cancer population showed that lower CC was strongly associated with malnutrition and risk of death (25). The lower CC may reflect the frailty of the patient to some extent. A study in older adults living in the community suggests that CC may be positively associated with better physical performance and lower frailty index (26). Therefore, CC may be an effective tool that can guide public health policy development. However, the evidence on the association between TC and all-cause and CVD mortality is insufficient. Our study shows that in the general population, higher TC is associated with lower all-cause and CVD mortality.

The following reasons may explain why all-cause mortality and CVD mortality are positively correlated with parameters measuring central obesity, and negatively correlated with parameters measuring extremity obesity. First, visceral fat accumulation may be one reason. The risk of obesity largely depends on the amount of visceral fat (27). Previous studies have reported that excessive visceral fat content is strongly associated with an increased risk of metabolic cardiovascular risk factors. The accumulation of visceral fat is often manifested as central obesity, which is an essential feature of metabolic disease. Therefore, larger WC may be associated with increased all-cause and CVD mortality (28, 29). Second, in individuals with a certain BMI, a larger AC, CC or TC may be associated with a smaller WC, which may be associated with a lower risk of all-cause and CVD mortality. Third, femoral fat has an improving effect on metabolism. Studies have shown that compared with the abdominal fat pool, the femoral fat pool is more passive and can store fatty acids for a long time to maintain its protective properties. Femoral fat also captures excess fatty acids, thereby preventing the adverse effects associated with ectopic fat precipitation. Third, in contrast to the effect of adipose tissue, increased muscle tissue content is associated with improved metabolic status, while a lack of muscle content may lead to the loss of its protective effect against adverse health outcomes (30–32). Therefore, a larger extremity circumference may mean more muscle content and a stronger protective effect on the body. In addition, the greater the AC, the better the quality of life, the lower the probability of accidental injury.

Our results may have important public health and clinical value. Obesity affects hundreds of millions of people around the world and has been specifically recognized as a disease in countries such as the United States, Germany and Canada (33). Therefore, it is important to develop strategies to prevent obesity in the public health field. Although computed tomography (CT), magnetic resonance imaging (MRI), and other auxiliary devices are recommended for the determination of fat content, they are difficult to be routinely used in epidemiological studies due to the high cost, the need for professional operators, and the risk of radiation exposure (34). Therefore, there is a need for a more convenient method to assess body fat than auxiliary medical devices. Our results show that all-cause and CVD mortality are positively associated with measures of central obesity and negatively associated with measures of extremity obesity, and that AC, CC, and TC can be used as potential tools to measure prognosis in the general population.

### 4.1. Strengths and limitations

Anthropometric parameters were measured by trained professionals, not self-reported, and are easy to measure, so our results may have general applicability. However, our study also has the following limitations: First, data on anthropometric parameters were obtained at enrollment, and the physical characteristics of the population may change over time. Therefore, we cannot rule out misclassification due to changes in body shape during follow-up. However, in epidemiological research, the use of baseline data is a widely accepted method (12, 13). Second, we adjusted for many covariates in Model 2, but since many factors may affect the mortality of the population, there may be additional confounders that are not adjusted. Third, we included only the US population, which may limit the general applicability of our results.

## 5. Conclusions

The cohort study compared the association between one measure of central obesity (WC) and three measures of extremity obesity (AC, CC, and TC) with all-cause and CVD mortality. The most prominent finding of this study was that increases in WC were associated with increased all-cause mortality and CVD mortality, while increases in AC, CC, and TC were associated with reduced all-cause and CVD mortality. Therefore, AC, CC, and TC may be used as potential tools to measure prognosis in the general population. Future large sample sizes, well-designed prospective studies should assess the exact role of extremity circumference in predicting the risk of CVD.

## Data Availability

The original contributions presented in the study are included in the article/Supplementary Material, further inquiries can be directed to the corresponding author.
